# Molecular epidemiology reveals distinct lineages and genomic profiles of avian pathogenic and commensal Escherichia coli in broiler flocks

**DOI:** 10.1099/mgen.0.001797

**Published:** 2026-07-23

**Authors:** Homayoon Davam, Désirée S. Jansson, Emma Nord, Peter Halvarsson, Robert Söderlund, Jesper Rydén, Ingrid Hansson

**Affiliations:** 1 Department of Animal Biosciences, Swedish University of Agricultural Sciences, SE-750 07 Uppsala, Sweden; 2 Department of Clinical Sciences, Swedish University of Agricultural Sciences, SE-750 07 Uppsala, Sweden; 3 Department of Microbiology, Swedish Veterinary Agency, SE-751 89 Uppsala, Sweden; 4 Department of Energy and Technology, Swedish University of Agricultural Sciences, SE-750 07 Uppsala, Sweden

**Keywords:** avian pathogenic *Escherichia coli* (APEC), colibacillosis, ColV plasmids, poultry, virulence-associated genes, whole-genome sequencing (WGS)

## Abstract

*Escherichia coli* is predominantly an intestinal commensal; however, avian pathogenic *E. coli* (APEC) causes colibacillosis in poultry. The APEC pathotype lacks a clear genetic definition, further complicated by its opportunistic nature. To compare the genomic characteristics of avian pathogenic and commensal *E. coli*, isolates from diseased and healthy broiler flocks in Sweden were analysed, collected between 2022 and 2024. Clinical isolates (*n*=202) were collected at necropsy from 40 flocks during colibacillosis outbreaks, and non-clinical isolates (*n*=109) were obtained from litter using sock sampling in 60 unaffected flocks. Whole-genome sequencing was performed to determine sequence types (STs), serotypes, phylogroups, virulence-associated genes (VAGs) and to identify ColV plasmids. A five-gene APEC marker panel targeting plasmid-associated virulence genes (*iutA, hlyF, iss, iroN* and *ompT*) was used to classify isolates as APEC or non-APEC, and high-risk clones were identified according to the APECtyper scheme. Clinical isolates comprised 22 STs and 25 serotypes and were dominated (59%) by the ST23 O78:H4 clone within phylogroup C. Non-clinical isolates were more diverse (44 STs, 67 serotypes), primarily within phylogroups A (48%) and B1 (33%), with no clone predominating. Clinical isolates carried significantly more VAGs (*P*<0.001). Overall, 97% of clinical isolates were identified as APEC, all of which carried a ColV plasmid. Among non-clinical isolates, 28% were APEC, of which 80% were ColV-positive. However, clinical APEC isolates carried significantly more ColV-associated virulence gene clusters than non-clinical APEC isolates (*P*<0.001). Only 5% of non-APEC isolates were ColV-positive. High-risk clones were restricted to clinical APEC isolates (63%). These findings indicate that colibacillosis in Swedish broilers was largely driven by a dominant APEC clone during the study period, highlighting the need for coordinated surveillance and targeted control of high-risk clones. The presence of VAG reservoirs among isolates from unaffected flocks, together with the limitations of marker-based APEC typing, supports integrated frameworks combining lineage, VAG profiles and plasmid content for more reliable APEC identification and pathogenicity assessment.

**Impact Statement**

The absence of a clear genomic definition of avian pathogenic *Escherichia coli* (APEC), combined with its opportunistic nature, limits effective surveillance and control of colibacillosis in poultry. Using whole-genome sequencing, this study demonstrated that colibacillosis in Swedish broilers was largely associated with a dominant ST23 O78:H4 clonal lineage, emphasizing the importance of clonal expansion in the epidemiology of colibacillosis. Substantial differences in population structure and virulence-associated gene (VAG) profiles were identified between clinical and non-clinical isolates. Clinical isolates were characterized by more conserved and complete virulence repertoires, while the presence of VAGs and ColV plasmids in non-clinical isolates highlighted the role of commensal populations as reservoirs of virulence determinants. These findings underscore the limitations of marker-based APEC classification and indicate that APEC is more accurately interpreted through integrated genomic frameworks. This enables improved identification of disease-associated *E. coli* and supports targeted control of colibacillosis in poultry production systems through measures such as early detection and tracking of dominant clones, guiding the selection of vaccine strains and distinguishing clonal dissemination from sporadic opportunistic infections.

## Data Summary

The authors confirm that all supporting data, code and protocols have been provided within the article or through supplementary data files. Raw sequencing data have been deposited in the European Nucleotide Archive under BioProject accession no. PRJEB89812 and are publicly available at https://www.ebi.ac.uk/ena/browser/view/PRJEB89812. Accession numbers for individual isolates are provided in Table S1, available in the online Supplementary Material.

## Introduction

Most *Escherichia coli* strains are commensals that colonize the intestinal tract of mammals and birds, whereas avian pathogenic *E. coli* (APEC), a subgroup of extraintestinal pathogenic *E. coli* (ExPEC), is a major cause of avian colibacillosis [1, 2]. Colibacillosis is among the most frequently reported bacterial infections in poultry worldwide and results in impaired welfare and substantial economic losses [3, 4]. It manifests as diverse systemic and local gross lesions, such as omphalitis, airsacculitis, perihepatitis, pericarditis and cellulitis [4].

Despite extensive research, colibacillosis remains difficult to control, as preventive measures are only partially effective and outbreaks continue to occur. While certain APEC lineages are strongly linked to colibacillosis as primary pathogens, *E. coli* isolated from diseased birds are not always classified as APEC [5]. Colibacillosis largely results from opportunistic infections, and both APEC and some non-APEC strains can cause disease under predisposing conditions such as immunosuppression, co-infections or stress [6]. Consequently, clinical signs alone are insufficient to distinguish commensal from pathogenic *E. coli*, and accurate differentiation requires molecular characterization [6, 7]. Classification of APEC has often relied on combinations of specific virulence-associated genes (VAGs). These genes enhance pathogenic potential through various mechanisms, including adhesion, invasion, colonization, serum resistance, iron acquisition and toxin production [8]. Several studies have proposed different VAG panels for classifying strains as APEC, commonly including *sitA*, *iss*, *iucD*, *hlyF*, *ompT*, *iroN*, *iutA*, *irp2*, *papC*, *cvaC* and *tsh* [9–11]. Many of these genes are encoded on large plasmids, particularly ColV plasmids, which play a key role in enhancing extraintestinal fitness and the disease-causing ability of *E. coli* [12, 13]. Nevertheless, the APEC pathotype remains unresolved and lacks a universally accepted definition. Although several VAGs are enriched among APEC strains [14], no VAG combination is consistently present across the APEC population [6]. Moreover, genes typically associated with APEC plasmids can also be abundant in commensal *E. coli* isolates [5, 7, 15]. Together, these patterns illustrate the limitations of relying solely on VAG panels and underscore the value of integrated genomic approaches for understanding APEC and its pathogenic potential. Beyond VAG content, population structure and lineage-based characterization provide complementary insights into APEC populations. Despite considerable genetic heterogeneity, certain APEC lineages are consistently associated with colibacillosis, suggesting that they possess genetic backbones that facilitate the acquisition and expression of virulence determinants [6, 16, 17]. Historically, *E. coli* has been classified into serogroups based on the somatic O-antigen, which reflects antigenic diversity and is often associated with the ecological and epidemiological distribution of isolates [18]. Serotypes O1, O2 and O78 are most commonly associated with avian colibacillosis and are often regarded as dominant APEC serotypes [16, 19]. Despite substantial genomic plasticity, limited chromosomal recombination in *E. coli* preserves a sufficiently clonal population structure to allow classification into broad evolutionary lineages [20]. Clermont *et al*. [21] developed a quadruplex PCR method for assigning *E. coli* isolates to seven phylogroups (A, B1, B2, C, D, E and F) [21], later expanded to eight with the addition of phylogroup G [22]. Phylogroups A and B1 are predominantly associated with commensal *E. coli*, whereas B2 and D often include ExPEC lineages, and APEC isolates are most frequently classified within phylogroups C and G [5, 16, 22, 23]. Another widely used typing method is multilocus sequence typing (MLST), which assigns isolates to sequence types (STs) based on allelic variation in seven conserved housekeeping genes, enabling high-resolution clonal analysis and epidemiological tracking [24]. Several genotypes are frequently reported among APEC, including ST23 in phylogroup C; ST117 in phylogroup G; and ST95, ST140 and ST428/ST429 in phylogroup B2 [16]. Increased accessibility of whole-genome sequencing (WGS) has transformed *E. coli* characterization by enabling established typing methods and virulence profiling to be integrated *in silico*. This approach provides a robust framework for assessing genetic variation among isolates and supports high-resolution tracking of their evolutionary and epidemiological dynamics [25]. Broiler production in Nordic countries (Denmark, Finland, Iceland and Norway) partly relies on imports of Swedish broiler breeders (supplied as hatching eggs or day-old chicks), which originate from grandparent stock imported into Sweden from Scotland [26]. Between 2014 and 2016, a notable increase in colibacillosis was reported on broiler farms in Denmark, Finland and Norway. WGS analyses revealed a diverse *E. coli* population with ST117 O78:H4 as the predominant lineage in affected flocks, and these closely related strains were suggested to have spread vertically through the broiler breeding pyramid [26, 27]. Similarly, transmission of extended-spectrum cephalosporin-resistant *E. coli* from imported breeding stock through the Swedish production pyramid has been documented [28]. Together, these findings emphasize the need for coordinated, WGS-based epidemiological investigations to identify dominant APEC lineages driving colibacillosis outbreaks. However, genomic and epidemiological data on colibacillosis-associated *E. coli* in Swedish broiler production remain limited, and comparative analyses of APEC and commensal populations are scarce. This study aimed to characterize the genomic diversity of *E. coli* in Swedish broiler production by comparing isolates from flocks with colibacillosis (clinical isolates) with those from flocks without clinical signs of disease and elevated mortality (non-clinical isolates). STs, serotypes, phylogroups, VAGs and ColV plasmid repertoires were analysed to define population structure, assess clonality and identify dominant APEC clones associated with colibacillosis in Swedish broiler flocks.

## Methods

### Study population and flock classification

Clinical and non-clinical *E. coli* isolates were collected from conventional broiler farms across Sweden. All chickens were of the Ross 308 strain, and sampling was conducted across flocks of different ages between November 2022 and May 2024. All participating farms were affiliated with the Swedish Poultry Meat Association, which represents ~98% of broiler production in the country. A total of 39 out of 109 registered conventional broiler farms (36%) were sampled in this study. During the study period, ~111 million broilers were produced annually in Sweden [29].

Clinical isolates were obtained from 40 broiler flocks on 26 farms. These flocks had elevated mortality over several consecutive days and were reported by farmers or field veterinarians as suspected cases of colibacillosis. Colibacillosis was subsequently confirmed at necropsy if more than 50% of chickens submitted for examination exhibited gross lesions consistent with colibacillosis, in combination with isolation of *E. coli* from affected organs. These flocks are hereafter referred to as ‘colibacillosis flocks’.

Non-clinical isolates were collected from 60 flocks across 26 farms where farmers had not observed an increase in mortality. Flocks were included in this category if chickens appeared clinically healthy and showed no increase in mortality during the 7 days prior to sampling. These flocks are hereafter referred to as ‘healthy flocks’.

Thirteen farms were represented in both clinical and non-clinical categories, but samples from these farms were obtained from different flocks and, except in one case, from separate production cycles. Flocks were additionally classified into three groups based on sampling age: early-stage (1–7 days), mid-stage (8–26 days) and late-stage (27–35 days) (Table 1).

**Table 1.** Summary of *E. coli* isolates (*n*=311) from Swedish broiler flocks included in the study

	Colibacillosis flock (*n*=40)	Healthy flock (*n*=60)
Number of farms	26	26
Early-stage flocks (1–7 days)	15 (76 isolates)	4 (7 isolates)
Mid-stage flocks (8–26 days)	6 (30 isolates)	29 (50 isolates)
Late-stage flocks (27–35 days)	19 (96 isolates)	27 (52 isolates)
Source of isolates	Liver (112), cellulitis (33), spleen (27), yolk sac (27), pericardial sac (2), joint capsule (1)	Litter (109)
Total number of isolates	202	109

### Post-mortem examination and sampling

From each colibacillosis flock, five to ten birds – either recently dead or euthanized for welfare reasons in accordance with farm protocols – were transported to the Swedish University of Agricultural Sciences (SLU) or the Swedish Veterinary Agency (SVA) for necropsy. A total of 344 chickens underwent post-mortem examination following a standardized in-house necropsy protocol based on the guidelines of Brugère-Picoux *et al*. [30]. To ensure consistency, all necropsies were performed by the same veterinarian throughout the study, and body condition and gross findings were recorded for each chicken. Chickens showing gross lesions characteristic of colibacillosis and minimal autolysis were selected for sampling. Of these, samples from 141 chickens were included in the study: 53 from early-stage, 22 from mid-stage and 66 from late-stage flocks. Sampling was conducted using sterile instruments and swabs (Copan Diagnostics, Inc., Murrieta, CA, USA). Following external examination, the subcutaneous tissue was examined first, and when cellulitis was observed, the lesion was swabbed. The body cavity was then opened, and the liver parenchyma and spleen were sampled by swabbing the incised tissue with a sterile scalpel. In chickens younger than 7 days, the yolk sac was also sampled.

From healthy flocks, faecal samples were collected from the litter using boot sock sampling. This non-invasive approach was used to avoid animal handling and minimize potential welfare impacts. Boot sock sampling has been demonstrated to provide a representative measure of intestinal carriage of bacteria in chickens and has been successfully used for sampling *E. coli* [31], *Campylobacter* spp*.* [32] and for regulatory screening of *Salmonella* spp. in broilers within the EU [Regulation (EC) No 646/2007]. Each sock (Danafast 7.5, Tubular Retention Bandage, Mediplast AB, Malmö, Sweden) was pre-moistened with 30 ml of buffered peptone water (BPW) (Oxoid, Basingstoke, UK) and placed over boots. Sampling was performed by walking across the litter to cover the floor area of the broiler house, ensuring full contact with faeces by periodically rotating the socks around the boots. Following sampling, the socks were transferred to plastic stomacher bags and transported at ambient temperature to SLU for bacteriological analysis.

### Bacteriological analyses and selection of isolates

Swabs from necropsied chickens were directly streaked onto 5% bovine blood agar (BA) plates (SVA, Uppsala, Sweden) and incubated at 37 ± 1 °C for 18–24 h. Sock samples were enriched in 90 ml of BPW, homogenized in a stomacher for 1 min and incubated under the same conditions. After enrichment, ~20 µl of the suspension was plated onto MacConkey agar plates (SVA, Uppsala, Sweden) and incubated at 37 ± 1 °C for 18–24 h. Two presumptive *E. coli* colonies were selected from each plate based on colony morphology and confirmed using MALDI-TOF MS (MALDI Biotyper, Bruker Daltonics, Germany). Confirmed isolates were re-cultured on BA to ensure purity and incubated at 37 ± 1 °C for 18–24 h. Pure isolates were stored at −70 °C in brain–heart infusion broth (CM1135; Oxoid) supplemented with 15% glycerol.

In total, 311 *E. coli* isolates were selected for inclusion in the study (Table 1). From each colibacillosis flock, four to eight isolates were chosen, yielding 202 isolates. Isolates from the liver were primarily selected due to the organ’s larger size and easier accessibility during necropsy, which helps reduce the risk of contamination during sampling. From each healthy flock, one or two isolates were selected, resulting in 109 isolates.

### Whole-genome sequencing and bioinformatics analyses

All 311 isolates were subjected to WGS. DNA extraction was conducted using magnetic-particle technology on the EZ1 Advanced XL instrument with the EZ1 DNA Tissue Kit (Qiagen, Hilden, Germany), following the manufacturer’s instructions. DNA concentrations were quantified with the Qubit dsDNA High Sensitivity Assay Kit on a Qubit 2.0 Fluorometer (Invitrogen, Carlsbad, CA, USA). Libraries were prepared using the Nextera XT DNA Library Preparation Kit (Illumina, San Diego, CA, USA), and library quality was assessed with High Sensitivity D1000 ScreenTape on the 4150 TapeStation System (Agilent Technologies, Santa Clara, CA, USA). Prepared libraries were submitted to SciLifeLab Clinical Genomics (Solna, Stockholm) and sequenced on an Illumina NovaSeq X instrument, generating 2×150 bp paired-end reads. Sequencing reads were quality-checked and trimmed with fastp v0.23.4 [33], downsampled to ~100× coverage using BBMap reformat v39.09 [34], and assembled *de novo* with Unicycler v0.5.1 [35]. Trimmed reads were screened for potential contamination using Kraken2 v2.1.3 [36], and assembly quality was evaluated with QUAST v5.0.2 [37]. STs were determined using FastMLST v0.0.16 [38], serotypes with SerotypeFinder v2.0.1 [18], and phylogroups with ClermonTyping v24.02 [39]. Isolates that could not be assigned an ST because they contained alleles absent from the MLST database were submitted to EnteroBase [40] for typing. VAGs were identified using VirulenceFinder v2.0.4 with database version 2.0.1 [41, 42]. blast+v2.17.0[43] was used to screen genome assemblies for the primer sequences of the five-gene virulence marker panel [10]. Core genome MLST (cgMLST) analysis was performed in Ridom SeqSphere+v10.5.5 [44] using the *E. coli* cgMLST v1 scheme available from cgmlst.org. An Unweighted Pair Group Method with Arithmetic Mean (UPGMA) tree was generated in Ridom SeqSphere+, and a minimum spanning tree (MST) was calculated in GrapeTree v2.1 [45] using the MSTreeV2 algorithm. Clusters were defined as groups comprising ≥5 isolates with a maximum pairwise distance of 10 cgMLST allelic differences. One sample with <95% of cgMLST alleles was excluded from the trees. For all analyses, default parameters were used, with some exceptions listed in Table S2.

### APEC typing and ColV-associated virulence gene profiling

Isolates were classified as APEC or non-APEC based on the presence of the five-gene virulence marker panel [10], comprising the plasmid-associated genes *iutA, hlyF, iss, iroN* and *ompT* (*ompTp*). Isolates carrying at least four of these genes were classified as APEC. High-risk clones were identified according to the classification scheme implemented in APECtyper [5]. As defined by Johnson *et al*. [5], isolates were classified as high-risk if they belonged to one of the dominant high-virulence STs (ST23, ST131, ST355 or ST428) or to serogroup O78, irrespective of their APEC classification.

ColV-associated genes were identified based on homology to the reference ColV plasmid pAPEC-O2-ColV [46] and grouped into six functional modules according to established operon structures and functionally related gene clusters described by Liu *et al*. [47] (Table 2). Isolates were classified as ColV-positive if they carried at least one gene from four or more of the six ColV-associated virulence modules.

**Table 2.** ColV-associated virulence modules and their constituent genes used to define ColV plasmid carriage in *E. coli* isolates from Swedish broiler flocks

Module*	Gene included	Function†
Colicin V (ColV operon)	*cvaA, cvaB, cvaC, cvi*	Colicin V production, secretion and immunity; enhances competitive fitness
Ets transporter	*etsA, etsB, etsC*	ATP-binding cassette transporter; likely contributes to within-host fitness
Outer membrane virulence factors	*hlyF, ompTp*	*hlyF* promotes outer membrane vesicle production; *ompTp* degrades host antimicrobial peptides
Salmochelin siderophore (Salmochelin operon)	*iroB, iroC, iroD, iroE, iroN*	Salmochelin-mediated iron acquisition
Aerobactin siderophore (Aerobactin operon)	*iucA, iucB, iucC, iucD, iutA*	High-affinity iron acquisition via aerobactin; supports growth in iron-limited environments
Sit metal transport	*sitA, sitB, sitC, sitD*	Metal transporter; contributes to metal acquisition

*Modules defined according to Liu *et al.* [47].

†Functions summarized based on Lian *et al.* [63].

### Statistical analysis

Statistical analyses were designed to compare the prevalence of VAGs and ColV-associated virulence modules between clinical and non-clinical isolates while accounting for clustering at the flock level. Analyses were conducted using R version 4.4.2 [48, 49]. For gene-level comparisons, VAGs with low prevalence were defined as those present in less than 10% of all isolates and were excluded from mixed-effects modelling unless they exceeded 10% in either the clinical or non-clinical group and showed a statistically significant difference between groups (*P*<0.05). VAGs exhibiting complete separation (i.e. present exclusively in one group) were classified as sparse and were also excluded from mixed-effects modelling. Genes with sufficient variation were analysed using generalized linear mixed-effects models (GLMMs) with a binomial distribution (gene presence/absence) and logit link, including group (clinical vs non-clinical) as a fixed effect and flock as a random intercept, implemented using the lme4 package (version 1.1.36) [49]. Sparse genes were analysed using contingency-table methods, applying the Cochran–Mantel–Haenszel test when stratification was required, and Fisher’s exact test otherwise. All *P*-values were adjusted for multiple testing using the Benjamini–Hochberg false discovery rate procedure, and adjusted *P*-values (*P*<0.05) were used for inference.

Differences in the average number of VAGs between clinical and non-clinical isolates were analysed using Gaussian GLMMs with group as a fixed effect and flock as a random intercept. The average number of ColV-associated virulence modules, treated as a bounded count outcome (modules in Table 2, range 1–6), was analysed using binomial GLMMs with the same fixed- and random-effects structure. Model fit and assumptions were evaluated using simulated residuals, allowing visual inspection and formal testing for overdispersion.

## Results

### Post-mortem findings

Among all necropsied chickens (*n*=344), 57% were male and 43% female. The most frequently observed gross lesions were pericarditis, perihepatitis, hepatomegaly and splenomegaly (Table 3). Yolk sac infection was common in chickens from early-stage flocks, whereas cellulitis was not observed in chickens younger than 27 days. In affected chickens, cellulitis lesions ranged from 10 to 70 mm in diameter.

**Table 3.** Distribution of the most common gross lesions among necropsied chickens from Swedish broiler flocks experiencing elevated mortality due to colibacillosis, stratified by age group. Percentages represent the proportion of chickens within each group in which the lesion was observed. A single chicken could exhibit more than one lesion

Lesion	All chickens (*n*=344) (%)	Early-stage flock (1–7 days; *n*=125) (%)	Mid-stage flock (8–26 days; *n*=57) (%)	Late-stage flock (27–35 days; *n*=162) (%)
Pericarditis	46	40	47	50
Perihepatitis	42	38	42	45
Hepatomegaly	35	30	33	38
Splenomegaly	27	22	26	32
Yolk sac infection	^–*^	47	5	–
Cellulitis	–	–	–	28

*The symbol ‘–" indicates that the lesion was not observed in the corresponding age group or that an overall percentage was not applicable for all chickens.

### Whole-genome sequencing and bioinformatic analyses

#### Sequence types and serotypes

Across all 311 isolates, 58 STs and 86 serotypes were identified. Clinical isolates (*n*=202) comprised 22 STs and 25 serotypes compared with 44 STs and 67 serotypes among non-clinical isolates (*n*=109).

**Fig. 1.**
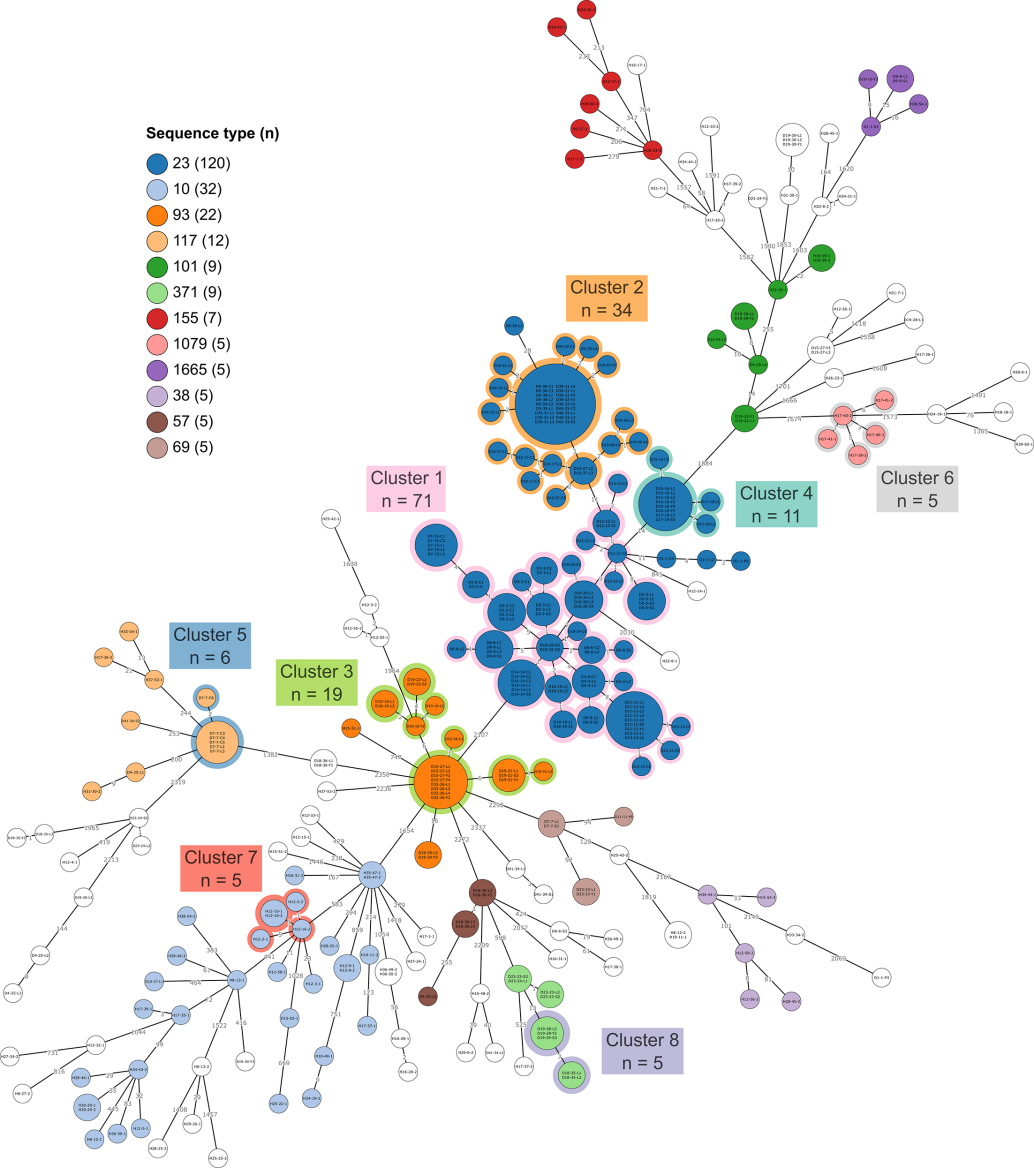
MST based on cgMLST of *E. coli* from Swedish broiler flocks (2022–2024). The tree includes 201 clinical isolates from flocks with colibacillosis and 109 non-clinical isolates from flocks without clinical signs. Each node represents a unique cgMLST allelic profile, with node sizes proportional to the number of isolates. Nodes are coloured by ST for STs comprising ≥5 isolates; remaining STs are shown in grey. Clusters comprising ≥5 isolates (outlined in colour) were defined using a maximum pairwise distance of 10 allelic differences, and cluster sizes are indicated (*n*=X). Edge lengths are scaled logarithmically, and numbers on edges indicate pairwise allelic differences. Labels within nodes encode isolate metadata as follows: D=clinical (diseased flock) and H=non-clinical (healthy flock), followed by farm ID–flock ID. Clinical isolates additionally include sampling site (L, liver; S, spleen; C, cellulitis lesion; Y, yolk sac; P, pericardial sac; J, joint capsule) and a numeric identifier indicating the individual chicken sampled within the flock. For non-clinical isolates, the final number denotes the isolate identifier within the flock.

Among clinical isolates, ST23 was predominant (59%) (Table 4). All ST23 isolates belonged to serotype O78:H4, and ST23 was detected in 25 of 40 colibacillosis flocks. The proportion of flocks in which ST23 was detected increased with broiler age (Table 4). The cgMLST analysis resolved ST23 isolates into three genetically closely related clusters, with only four ST23 isolates remaining outside these clusters (Fig. 1). ST93 was the second most frequent ST among clinical isolates (10%) and was largely confined to a single cgMLST cluster. Twenty-two colibacillosis flocks exhibited a single ST–serotype combination among all isolated *E. coli*, including 20 flocks dominated by ST23 O78:H4 and 2 by ST93 O5:H40. Among clinical isolates, more than half of the detected STs (12/22) and the majority of serotypes (16/25) were represented by only one or two isolates. From 57 chickens, multiple isolates were included (*n*=119). While most isolates from the same chicken shared an identical ST, divergent STs were detected in five chickens. In these chickens, three yolk sac and two spleen isolates differed from the liver isolate obtained from the same chicken. Among non-clinical isolates, ST10 was the most prevalent ST (28%) (Table 5), whereas no serotype predominated. The cgMLST analysis revealed a diverse population structure, with only two small clusters corresponding to ST10 and ST1079 (five isolates each) (Fig. 1). Of the 44 identified STs, 28 were represented by a single isolate, as were 47 of the 67 serotypes. Although no ST–serotype combination predominated among non-clinical isolates, the most frequent was ST10 O113:H4 (6%), followed by ST10 O145:H40, ST1079 O6:H49 and ST38 O153:H9, each detected in 5% of the isolates.

Nine STs and seven serotypes were shared between clinical and non-clinical isolates. Most of these were detected in only a single isolate in one of the groups. Exceptions included ST117 (eight clinical, four non-clinical) and ST101 (six clinical, three non-clinical). The only serotype shared by multiple isolates in both groups was O8:H8, detected in three clinical and two non-clinical isolates.

**Table 4.** The four most prevalent STs among 202 clinical *E. coli* isolates from 40 Swedish broiler flocks with colibacillosis

ST	No. of isolates (%)	No. of flocks where ST was identified		
		Early-stage (% of isolates)^*^	Mid-stage (% of isolates)	Late-stage (% of isolates)
**23**	120 (59)	4 (29)	4 (67)	17 (81)
**93**	21 (10)	5 (25)	1 (7)	–
**371**	9 (4)	5 (12)	–	–
**117**	8 (4)	–—	1 (3)	2 (7)

*Values in parentheses indicate the proportion of clinical isolates within each age group that belonged to the indicated ST. The symbol ‘–’ indicates that the ST was not detected in the corresponding age group.

**Table 5.** The four most prevalent STs among 109 non-clinical *E. coli* isolates from 60 Swedish broiler flocks without clinical signs of colibacillosis

ST	No. of isolates (%)	No. of flocks where ST was identified		
		Early-stage (% of isolates)	Mid-stage (% of isolates)^*^	Late-stage (% of isolates)
**10**	31 (28)	–	10 (26)	15 (35)
**155**	7 (6)	–	5 (10)	2 (4)
**38**	5 (5)	–	5 (10)	–
**1079**	5 (5)	–	–	3 (10)

*Values in parentheses indicate the proportion of non-clinical isolates within each age group that belonged to the indicated ST. The symbol ‘–’ indicates that the ST was not detected in the corresponding age group.

#### Phylogroup distribution

**Fig. 2.**
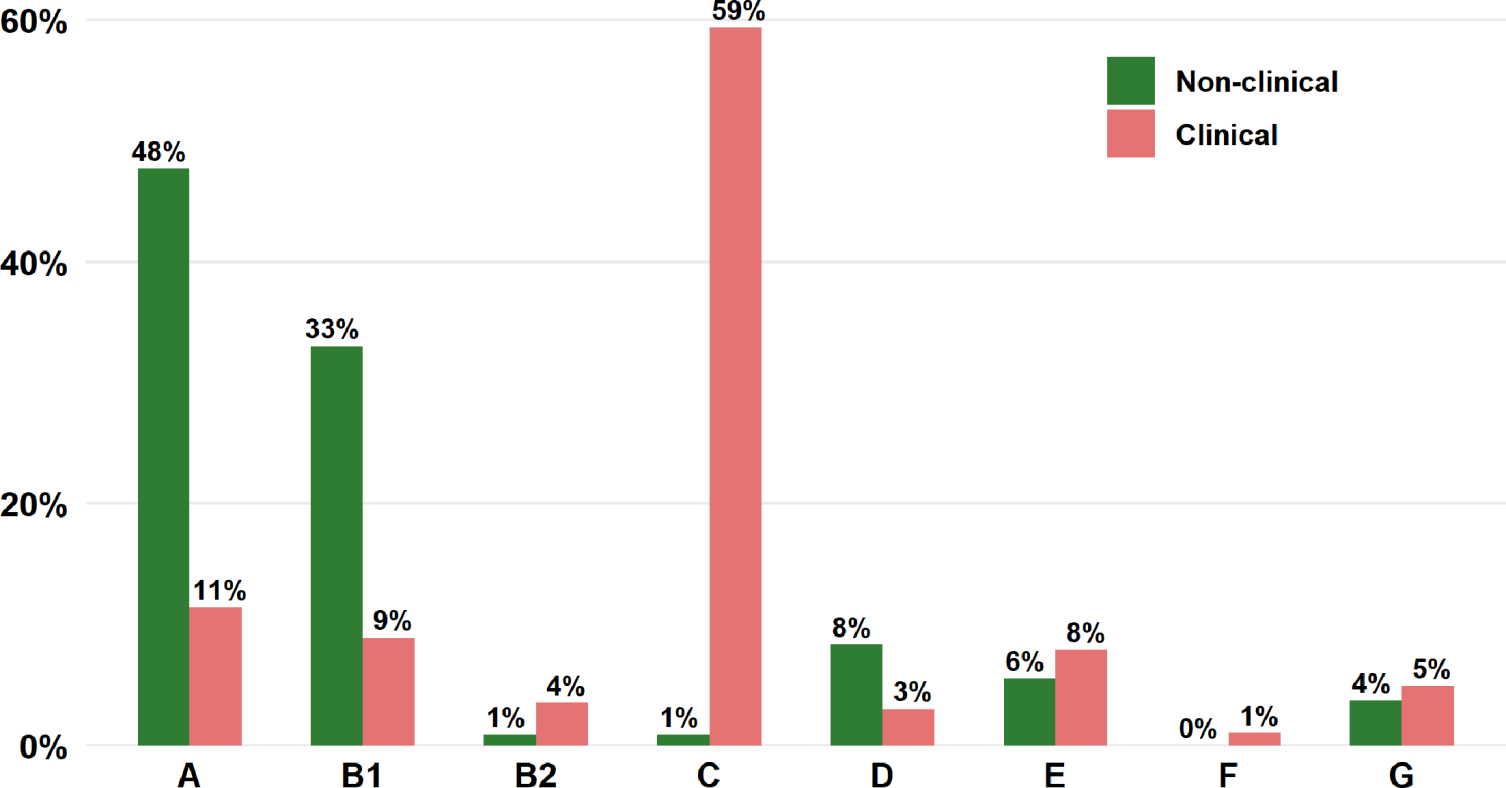
Distribution (%) of phylogroups among 202 clinical *E. coli* isolates from Swedish broiler flocks with clinical signs of colibacillosis and 109 non-clinical isolates from broiler flocks without clinical signs.

**Fig. 3.**
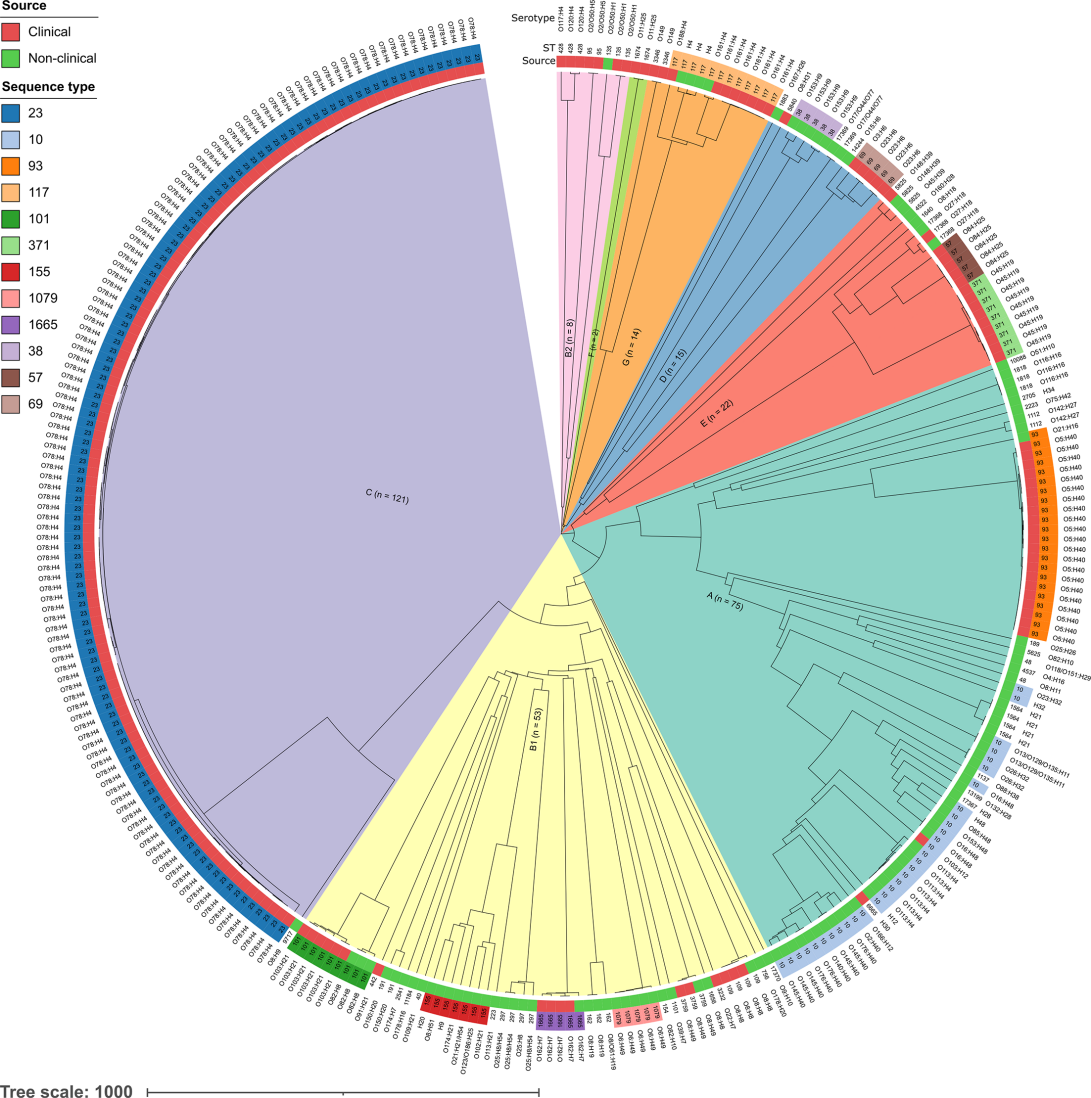
UPGMA hierarchical clustering based on pairwise cgMLST allelic differences of *E. coli* isolates from Swedish broiler flocks. The tree includes 201 clinical isolates from flocks with colibacillosis and 109 non-clinical isolates from flocks without clinical signs. Coloured sectors within the tree indicate phylogroups (A, B1, B2, C, D, E, F and G), with the number of isolates per phylogroup indicated (*n*=X). The first annotation ring denotes isolate source (red=clinical; green=non-clinical). The second ring indicates ST; STs comprising ≥5 isolates are coloured, and remaining STs are shown in grey. The outermost circle lists serotypes. The scale bar represents allelic differences.

The majority of clinical isolates were assigned to phylogroup C (Fig. 2), and all clinical isolates within this phylogroup were of the ST23 O78:H4 ST–serotype combination (Fig. 3). Among non-clinical isolates, ~81% belonged to phylogroups A and B1. Phylogroups B2, F and G each represented ≤5% of isolates in both groups.

#### Virulence-associated gene profiles

**Fig. 4.**
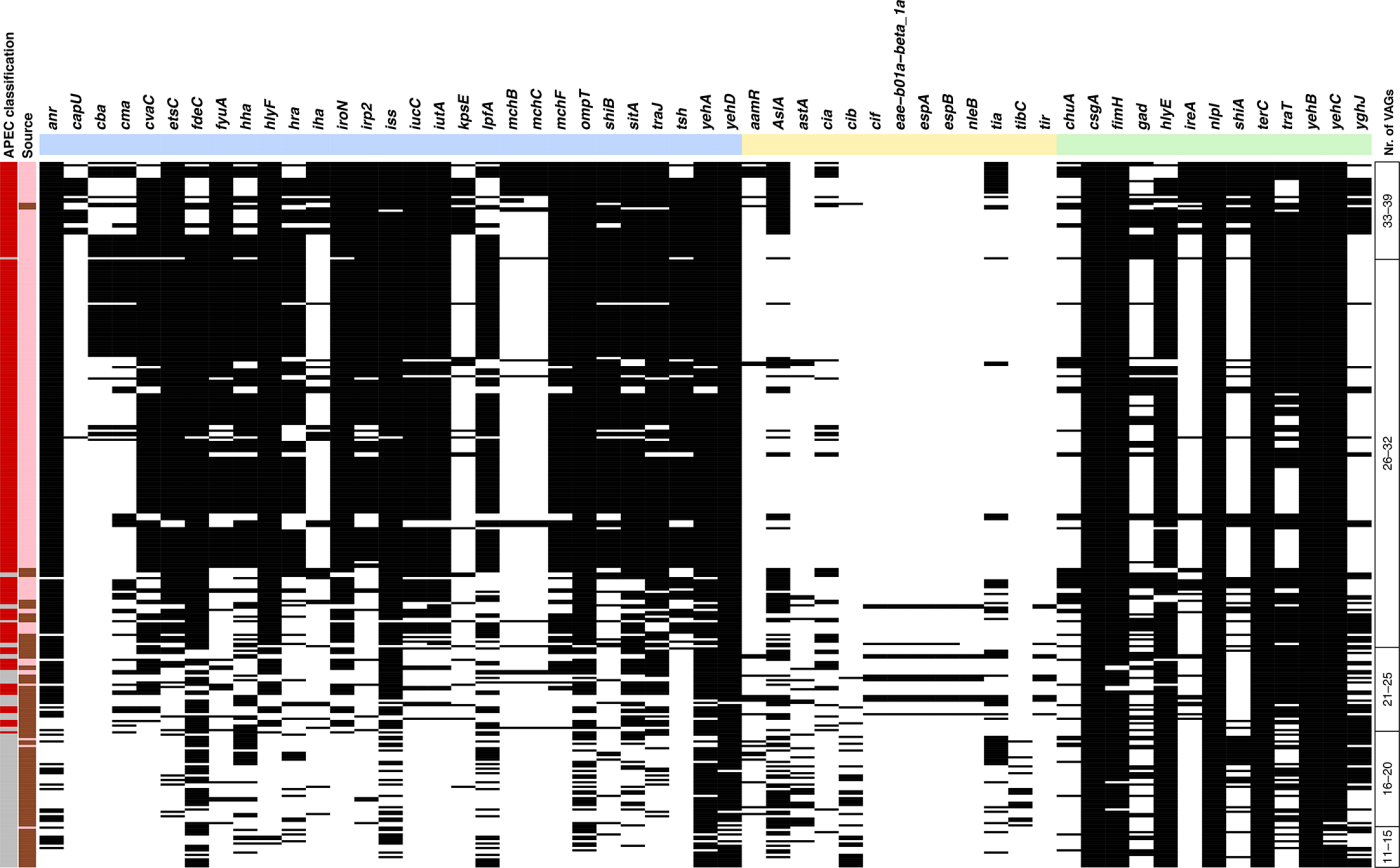
Distribution of 55 VAGs detected in 202 clinical *E. coli* isolates from Swedish broiler flocks with clinical signs of colibacillosis and 109 non-clinical isolates from flocks without clinical signs. Heatmap showing the presence (black) or absence (white) of VAGs per isolate (rows=isolates; columns=genes). Isolates are ordered by total VAG count, with gene names indicated above the heatmap. Brackets on the right denote ranges of the total number of VAGs per isolate. The first vertical bar on the left indicates APEC classification based on the five-gene marker panel (red=APEC; grey=non APEC), and the second bar denotes isolate source (pink=clinical; brown=non-clinical). The coloured band beneath the gene names indicates differential prevalence between groups: genes significantly more prevalent among clinical isolates (blue), significantly more prevalent among non-clinical isolates (yellow), and genes not significantly different between groups (green). Statistical significance was defined as adjusted *P*<0.05.

*In silico* analyses identified 105 VAGs across all isolates (Table S3). In this full panel, the mean number of VAGs per isolate was significantly higher among clinical isolates than among non-clinical isolates (32 vs 22 genes, *P*<0.001). After excluding VAGs with low prevalence, a panel of 55 VAGs was analysed, and the mean number of VAGs per isolate remained significantly higher among clinical isolates than among non-clinical isolates (30.7 vs 20.5 genes, *P*<0.001). Of these 55 VAGs, 42 differed significantly between groups (*P*<0.05), with 29 more prevalent among clinical isolates and 13 more prevalent among non-clinical isolates (Fig. 4).

#### APEC classification, high-risk clones and ColV plasmid distribution

Using the five-gene APEC virulence marker panel, 97% (196/202) of clinical isolates and 28% (30/109) of non-clinical isolates were classified as APEC (Table 6). Among clinical isolates, 184 carried all 5 genes and 12 carried 4, whereas the remainder carried 3 (*n*=3) or none (*n*=3). Among non-clinical isolates, 11 carried all 5 genes and 19 carried 4, whereas the remainder carried 3 (*n*=6), 2 (*n*=6), 1 (*n*=4) or none (*n*=63). ColV plasmids were detected in all clinical APEC isolates and 80% of non-clinical APEC isolates (Table S4). However, the mean number of ColV-associated virulence modules per isolate was significantly higher among clinical APEC isolates than among non-clinical APEC isolates (*P*<0.001). Among 85 non-APEC isolates from both clinical and non-clinical groups, only four were ColV-positive (one clinical and three non-clinical) (Table 6). High-risk clones, identified using the APECtyper scheme, were exclusively found within the clinical APEC group, including ST23 (*n*=120) and ST428 (*n*=3).

**Table 6.** APEC classification, distribution of VAGs, ColV plasmid presence, high-risk clone frequency and phylogroup distribution among 202 clinical *E. coli* isolates from Swedish broiler flocks with clinical signs of colibacillosis and 109 non-clinical isolates from flocks without clinical signs

Isolate	*n*	Mean VAG	ColV-positive* (%)	Mean ColV-associated module	High-risk† (%)	Phylogroup (%)							
						A	B1	B2	C	D	E	F	G
**Clinical APEC**	196	32.3	100	5.8	63	11	8	4	61	3	7	1	5
**Clinical non-APEC**	6	25	17	1.8	0	17	33	–^3^	–	–	33	–	17
**Non-clinical APEC**	30	26.7	80	4.9	0	27	43	3	3	10	–	–	14
**Non-clinical non-APEC**	79	20.2	4	0.6	0	56	28	–	–	8	8	–	–

*Isolates were classified as ColV-positive if they carried at least one gene from ≥4 of the six ColV-associated virulence modules.

†Isolates were classified as high-risk if they belonged to one of the dominant high-virulence STs (ST23, ST131, ST355 or ST428) or to serogroup O78. 3The symbol ‘–’ indicates that no isolates belonged to the corresponding phylogroup.

## Discussion

Defining the genetic boundaries between APEC and commensal *E. coli* remains a key obstacle to establishing a coherent genomic framework for the APEC pathotype and understanding the epidemiology of avian colibacillosis. This study provides a comparative genomic analysis of disease-associated (clinical) and non-disease-associated (non-clinical) *E. coli* isolates from broiler flocks, highlighting differences in population structure, VAG repertoires and plasmid content, and identifying the APEC lineages circulating in Swedish broilers.

The ST–serotype combination ST23 O78:H4 was the dominant clonal lineage in Swedish broilers with colibacillosis, consistent with its status as a major globally distributed APEC lineage [5, 16]. A clear regional concordance exists between our findings and recent genomic studies of *E. coli* in broilers with colibacillosis across the Nordic region, where the ST23 clone within serogroup O78 predominated in Finland [50], Norway [51] and Denmark [52]. This pattern is consistent with previous findings suggesting dissemination of APEC clones through integrated production systems [26] and is further supported by the Finnish study, in which affected flocks were epidemiologically linked through shared parent and grandparent flocks [50]. In addition, these findings support a regional clonal shift from the previously predominant ST117 O78:H4 reported between 2014 and 2016 [26, 50]. ST23 (phylogroup C) and ST117 (phylogroup G) belong to distinct phylogenetic lineages [25]. Although phylogroup C is closely related to phylogroup B1, it includes well-recognized APEC lineages such as ST23 O78 [21, 25]. The ST23 strains consistently carry the O78 antigen, whereas ST117 strains exhibit variable O-antigens while maintaining the H4 antigen [16]. These differences may influence ecological fitness and potentially affect lineage dominance. In our dataset, all ST23 isolates were O78:H4, whereas none of the ST117 isolates carried the O78 antigen and all encoded H4. Collectively, these findings emphasize the importance of coordinated genomic surveillance across interconnected poultry production systems.

Given the variability in how APEC is defined – from the presence of specific VAGs or pathogenic lineages to any *E. coli* recovered from diseased birds [5, 6] – we initially categorized isolates as clinical or non-clinical based on flock health status and isolation source. Isolates were then classified as APEC or non-APEC using the five-gene VAG marker panel [10], which has been reported to reliably distinguish APEC from avian faecal *E. coli* (AFEC) [53], and its widespread adoption facilitates comparison with other studies. As a subpathotype within ExPEC, APEC commonly resides in the broiler intestinal microbiota but can translocate across the intestinal barrier to cause systemic infection [15]. Consistent with this, clinical isolates from extraintestinal organs and lesions associated with colibacillosis were predominantly APEC (97%), whereas 28% of non-clinical isolates recovered from litter in healthy flocks were also classified as APEC. These findings further indicate that classification as APEC does not necessarily imply the ability to readily cause colibacillosis, as disease development is determined not only by bacterial pathogenic potential but also by host susceptibility, which may be influenced by predisposing factors [6]. Nevertheless, extraintestinal disease depends on the capacity of strains to enter and colonize extraintestinal sites, which is primarily linked to possession of specific VAGs [15]. A key finding in our study was that clinical and non-clinical isolates differed significantly in both average VAG count and virulence repertoire composition. Clinical APEC isolates exhibited the highest average VAG count, followed by non-clinical APEC isolates, whereas non-clinical non-APEC isolates had the lowest. While these patterns support enrichment of VAGs among disease-associated strains, VAG counts can be misleading, as many genes in VAG databases encode general fitness or colonization traits that are also common in commensal *E. coli* [7, 54]. In a comprehensive review comparing pathogenic and non-pathogenic *E. coli*, Ovi *et al*. identified a set of 10 key VAGs (*iroN, iutA*, *iucD, cvaC*, *ompTp*, *hlyF, tsh, iss, papG* and *papC*) frequently associated with APEC and proposed to play a central role in establishing systemic colibacillosis in chickens [55]. These genes span three principal functional categories: iron acquisition, secretion, and host colonization, host invasion and survival. In our dataset, these genes were significantly more prevalent in clinical than in non-clinical isolates, except for *papG* and *papC*, which were rare in both groups. When restricting the analysis to APEC-classified isolates, *tsh*, *iutA* and *iucD* were significantly more prevalent in clinical APEC than in non-clinical APEC isolates, whereas the remaining genes (*iss, iroN*, *ompTp, cvaC* and *hlyF*) were highly prevalent in both groups and did not differ significantly between them. Notably, all ST23 isolates carried the complete set of these eight VAGs, consistent with a conserved virulence repertoire that likely contributes to the dominance of this clonal lineage in colibacillosis cases. Together, these findings highlight that assessment of pathogenic potential should prioritize the composition and functional relevance of virulence repertoires rather than gene counts alone.

Many VAGs commonly associated with APEC are plasmid-encoded, including the key VAGs discussed above, with the exception of the *pap* genes [55]. Although the plasmid repertoire of *E. coli* is highly diverse, only a limited subset of plasmids encode virulence and fitness determinants in ExPEC [12]. Among these, ColV plasmids are regarded as major contributors to APEC pathogenicity. In our dataset, all clinical APEC isolates and 80% of non-clinical APEC isolates were ColV-positive, whereas ColV carriage was rare among non-APEC isolates. The strong overlap between APEC-classified and ColV-positive isolates was expected, as the APEC marker panel targets five VAGs encoded on ColV plasmids [10]. However, the APEC marker panel targets only a subset of ColV-associated virulence genes, and isolates may harbour partial or variant ColV-like virulence regions arranged in different combinations [15], without meeting the stricter definition of ColV carriage applied in this study. Our module-based analysis therefore provides an additional layer of resolution beyond marker-based APEC typing and simple presence/absence of ColV plasmids. Clinical APEC isolates carried, on average, significantly more ColV-associated modules than non-clinical APEC isolates. Notably, all ST23 isolates carried the complete set of six defined virulence modules. This distinction is biologically meaningful, as the mere presence of ColV-associated marker genes does not necessarily indicate equivalent pathogenic potential; rather, the gene content and functional completeness of ColV plasmids likely determine their contribution to disease. Such functional modularity helps explain why some non-clinical APEC isolates can exhibit relatively high VAG counts without causing colibacillosis.

The presence of APEC-classified and ColV-positive isolates in the non-clinical group supports the view that the intestinal microbiota acts as a reservoir of virulence determinants. Similarly, our parallel study of antimicrobial resistance in a subset of these isolates identified non-clinical isolates as reservoirs of resistance genes, whereas resistance among clinical isolates was negligible [56]. Horizontal gene transfer (HGT) enables the movement of mobile genetic elements between bacterial populations and is a major driver of *E. coli* evolution [57, 58]. Acquisition of virulence plasmids potentially enhances pathogenicity in previously commensal *E. coli* strains [46], enabling them to meet APEC classification criteria and, depending on host susceptibility, contribute to extraintestinal infection [54, 59]. However, carriage of genes primarily beneficial for extraintestinal adaptation imposes a fitness cost on the bacterium and may reduce its competitive advantage within the intestinal microbiota [15]. This may partly explain the enrichment of VAGs together with the near absence of resistance genes among clinical isolates [56], suggesting selection for lineages that retain VAGs without the additional burden of resistance determinants in Sweden’s low-antibiotic-use production system. In contrast, non-clinical isolates constitute a genetically heterogeneous reservoir of virulence and resistance genes. High bacterial density and genomic heterogeneity within the intestinal microbiota may facilitate HGT [60, 61], supporting the maintenance of mobile genetic elements. Interpretation of the non-clinical group, however, requires caution. Boot-sock sampling is intended to reflect flock-level intestinal shedding but may also capture strains from the broiler house environment, which can influence the observed genetic diversity. Despite this limitation, the contrast between isolate groups in this study remains clear. The predominance of ST23 strains, together with their conserved and complete ColV module profile, suggests that consistent extraintestinal disease emergence requires a compatible genomic background capable of maintaining and effectively expressing key VAGs associated with colibacillosis.

Although pathogenicity was not assessed in this study, our results are consistent with the ‘high-risk clone’ framework, in which a subset of APEC clones dominate colibacillosis in broilers [5]. The increased prevalence in late-stage flocks and the high within-flock clonality observed when ST23 was present further support the clonal expansion of highly adapted lineages. This aligns with studies indicating that highly virulent APEC strains become more prevalent in later production stages, displacing opportunistic strains [54]. In contrast, many flocks lacking ST23 were characterized by multiple low-frequency STs. Divergent STs were observed among isolates recovered from different organs in five chickens, which may indicate co-infection with distinct *E. coli* strains. Although contamination during sampling cannot be excluded, in four of these chickens, isolates from both organs were classified as APEC, whereas both isolates were non-APEC in the fifth chicken. Notably, none of these chickens originated from flocks in which ST23 was detected. Together, these observations support the view that once dominant clones become established, they can disseminate systemically and colonize multiple organs, whereas in their absence, disease may arise from diverse opportunistic strains, particularly under increased host susceptibility [62].

In conclusion, our genomic analysis indicates that colibacillosis in Swedish broilers is largely driven by a highly virulent ST23 O78:H4 clonal lineage. The overlap in virulence determinants between clinical and non-clinical isolates, together with the presence of ColV plasmids in some non-APEC isolates, underscores the limitations of single-marker definitions of the APEC pathotype. Rather, a more robust classification emerges from integrating lineage background (ST, serotype, phylogroup), VAG repertoire and plasmid composition. From an applied perspective, our findings support WGS-based surveillance strategies that prioritize the detection and tracking of dominant high-risk lineages across interconnected production systems. Such data can support targeted control measures by improving our understanding of factors contributing to the persistence and spread of colibacillosis and informing future disease prevention strategies, such as the selection of relevant vaccine strains.

## Supplementary material

10.1099/mgen.0.001797 Supplementary Material 1.

## Abbreviations

APEC: avian pathogenic *Escherichia coli*
BA: blood agar
BPW: buffered peptone water
cgMLST: core genome multilocus sequence typing
ExPEC: extraintestinal pathogenic *Escherichia coli*
GLMM: generalized linear mixed-effects model
HGT: horizontal gene transfer
MLST: multilocus sequence typing
MST: minimum spanning tree
SLU: Swedish University of Agricultural Sciences
ST: sequence type
SVA: Swedish Veterinary Agency
UPGMA: Unweighted Pair Group Method with Arithmetic Mean
VAG: virulence-associated gene
WGS: whole-genome sequencing
